# Physiological alterations of pineal recess crowding in symptomatic non-hydrocephalic pineal cysts

**DOI:** 10.1093/braincomms/fcad078

**Published:** 2023-03-17

**Authors:** Per Kristian Eide, Erika Kristina Lindstrøm, Are Hugo Pripp, Lars Magnus Valnes, Geir Ringstad

**Affiliations:** Institute of Clinical Medicine, Faculty of Medicine, University of Oslo, N-0316 Oslo, Norway; Department of Neurosurgery, Oslo University Hospital—Rikshospitalet, N-0424 Oslo, Norway; Institute for Cancer Genetics and Informatics, Oslo University Hospital, N-0424 Oslo, Norway; Oslo Centre of Biostatistics and Epidemiology, Research Support Services, Oslo University Hospital, N-0424 Oslo, Norway; Faculty of Health Sciences, Oslo Metropolitan University, N-0176 Oslo, Norway; Department of Neurosurgery, Oslo University Hospital—Rikshospitalet, N-0424 Oslo, Norway; Department of Radiology, Oslo University Hospital- Rikshospitalet, N-0424 Oslo, Norway; Department of Geriatrics and Internal Medicine, Sorlandet Hospital, N-4838 Arendal, Norway

**Keywords:** symptomatic pineal cysts, cerebrospinal fluid, glymphatic function, magnetic resonance imaging, pathophysiology

## Abstract

Pineal cysts are prevalent in the population. Due to more widespread use of magnetic resonance imaging, an increasing number of symptomatic patients with non-hydrocephalic pineal cysts are referred to neurologists and neurosurgeons. Currently, there is no generally accepted theoretical framework for linking symptoms to a pineal cyst. We have previously suggested that cyst-induced crowding of the pineal recess may affect venous runoff from the deep cerebral veins crossing the cyst. However, evidence underpinning this hypothesis is sparse. In the present study, we asked whether crowding of the pineal recess without imaging signs of hydrocephalus may be accompanied with alterations in blood flow of the internal cerebral veins, cerebrospinal fluid flow in the Sylvian aqueduct and cerebrospinal fluid-mediated tracer clearance from the brain along extravascular pathways (referred to as glymphatic function). This prospective, observational study included symptomatic individuals with non-hydrocephalic pineal cysts who underwent a standardized magnetic resonance imaging protocol (*n* = 25): Eleven patients were treated surgically with craniotomy and cyst extirpation and 14 individuals were managed conservatively without surgery. Our findings suggest that cyst-induced crowding of the pineal recess may have brain-wide effects: (i) There was a significant negative correlation between degree of crowding within the pineal recess and change in maximum venous flow velocity at the cyst, and a significant positive correlation between maximum venous flow velocity change at the cyst and net cerebrospinal fluid flow in the Sylvian aqueduct; (ii) increased degree of crowding in the pineal recess was accompanied by significantly impaired glymphatic enrichment in the cerebral cortex and subcortical white matter, indicative of a brain-wide effect in this cohort who also reported markedly impaired subjective sleep quality; (iii) there was a significant negative correlation between the apparent diffusion coefficient (suggestive of interstitial water content) within the thalamus and glymphatic enrichment of tracer and (iv) pineal recess crowding associated with symptoms. Comparison of the surgical cases [in whom 10/11 (91%) reported marked clinical improvement at follow-up] and the conservatively managed cases [in whom 1/14 (7%) reported marked clinical improvement at follow-up] showed differences in pre-treatment glymphatic tracer enrichment as well as differences in tracer enrichment in subarachnoid cerebrospinal fluid spaces. Taken together, we interpret these observations to support the hypothesis that cyst-induced crowding of the pineal recess without hydrocephalus may alter blood flow of the internal cerebral veins and cerebrospinal fluid flow and even cause brain-wide impairment of glymphatic transport with possible implications for cerebrospinal fluid transport of trophic factors such as melatonin.

## Teaser

Pineal cysts are usually asymptomatic. Eide *et al*. examined individuals with symptomatic non-hydrocephalic pineal cysts and provided evidence that crowding of the pineal recess is associated with changes in internal cerebral veins flow and cerebrospinal fluid flow and brain-wide alterations in glymphatic transport.

## Graphical Abstract

**Graphical Abstract:**
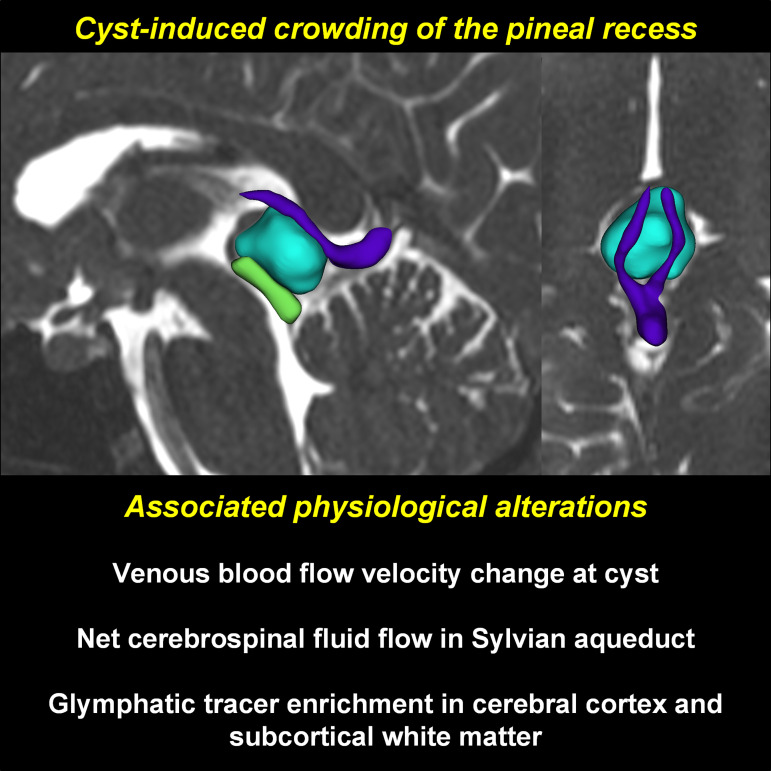

See Pickard and Santarius (https://doi.org/10.1093/braincomms/fcac096) for a scientific commentary on this article.

## Introduction

Pineal cysts are prevalent benign lesions of the pineal gland, occurring in at least 4% of the population,^1-3^ and usually with a benign natural course.^1^ A minor fraction present with hydrocephalus caused by compression of the tectal plate with secondary stenosis of the Sylvian aqueduct, sometimes combined with upward gaze palsy (Parinaud’s syndrome), which may require treatment.^4^ Due to the more widespread use of magnetic resonance imaging (MRI) in developed countries, an increasing number of patients are referred to neurologists and neurosurgeons for work-up of possibly symptomatic pineal cysts, even in the absence of hydrocephalus or gaze palsy. However, physiological effects that could link the cyst to symptoms have not been established, and there are currently no particular symptoms that can be specifically attributed to pineal cysts *per se*. Furthermore, the symptomatic picture can be complex, spanning from headaches to sleep disorders, fatigue and dizziness.

The vast majority of patients presenting with these symptoms and MRI findings of a non-hydrocephalic pineal cyst are managed conservatively, regardless of pineal cyst size.^1^ In selected cases, typically with larger cysts and a heavy symptom burden, surgical removal of the cyst is sometimes considered (Supplementary Fig. 1), which in some studies has been reported to improve symptoms.^5-7^ However, surgical treatment remains highly controversial,^6,8^ not least due to the paucity of controlled studies.

At present, there is a discrepancy between reports of patients experiencing marked clinical improvement after surgical cyst removal and our incomplete understanding of pathogenic events possibly evoked by a pineal cyst. Hence, there is an obvious need for a better characterization of effects exerted by pineal cysts both locally and on the brain as whole. In previous work, we have proposed that in some symptomatic cases with non-hydrocephalic pineal cysts, crowding of the pineal recess may affect the venous runoff from the internal cerebral veins when the pineal cyst dislocates the veins.^9,10^ Impaired venous runoff might in turn cause central venous hypertension directly propagating into deep cerebral structures like the thalamus and basal ganglia and also evoke an effect on intracranial pulsatile pressure. Indeed, we previously reported that MRI biomarkers of a crowded pineal recess associated with severity of symptoms,^9^ intracranial pressure scores^10^ and outcome of surgery.^7^ The present prospective and observational study was undertaken to explore a hypothesis (Supplementary Fig. 2) that pineal recess crowding from a non-hydrocephalic pineal cyst is accompanied with alterations in several physiological variables that can be directly assessed by MRI. We specifically addressed blood flow velocity changes in the internal cerebral veins, CSF flow velocity changes and alterations in glymphatic function. The glymphatic system is a brain-wide paravascular pathway for CSF-mediated brain nutrition and metabolic clearance, enhanced by sleep.^11^ Intriguingly, the primary role of the pineal gland is secretion of the sleep hormone melatonin.^12^ Possibly, reduced glymphatic function could be associated with impaired cerebral distribution of melatonin.

The results presented here suggest that pineal cysts with a size sufficient to cause crowding within the pineal recess evoke brain-wide physiological effects that may possibly associate with symptoms, even in the absence of hydrocephalus.

## Materials and methods

### Approvals

The present study was approved by The Regional Committee for Medical and Health Research Ethics (REK) of Health Region South-East, Norway (2015/96), The Institutional Review Board of Oslo University Hospital (2015/1868) and The National Medicines Agency (15/04932-7), and registered in Oslo University Hospital Research Registry (ePhorte 2015/1868). Patients were included after written and oral informed consent. The study was governed by ethical standards according to the Helsinki Declaration of 1975 (and as revised in 1983).

### Experimental design

A prospective and observational study design was followed, aiming at determining whether MRI markers of pineal recess crowding exerted by a pineal gland cyst are correlated with alterations in intracranial fluid flow (specifically blood flow of the internal cerebral veins and CSF net flow in the Sylvian aqueduct), tracer movement within CSF spaces and CSF-mediated tracer clearance from the brain along extravascular pathways (referred to as glymphatic function). The underlying hypothesis is that pineal cyst-induced crowding of the pineal recess leads to compromised venous runoff, increased interstitial water content and possibly altered glymphatic function (Supplementary Fig. 2).

### Patients with symptomatic non-hydrocephalic pineal cysts

Participants were symptomatic patients with non-hydrocephalic pineal cysts referred to the Department of Neurosurgery, Oslo University Hospital—Rikshospitalet, Oslo, Norway, from local neurological departments. They were included consecutively according to the study protocol. Inclusion criteria for participation were as follows: severe symptoms impacting quality of life and occupational capacity and MRI finding of a pineal cyst occupying the pineal recess in a patient with no other plausible causes of symptoms. Exclusion criteria were as follows: history of hypersensitivity reactions to contrast agents, history of severe allergy reactions in general, evidence of renal dysfunction and pregnant or breastfeeding women or individuals aged <18 or >80 years.

#### Assessment of symptom severity and outcome

The study protocol included standardized assessment of symptom severity according to our previously reported grading scale for assumed symptomatic non-hydrocephalic pineal cysts (Supplementary Table 1).^9^ Since there is no consensus which symptoms would be specific for non-hydrocephalic pineal cysts, though hypothesized being related to altered venous runoff, symptoms similar to those seen in central venous thrombosis were considered relevant.^13^

The clinical assessment preceded MRI acquisitions performed over a 3-day admission in the Department of Neurosurgery. Follow-up assessment of symptoms was done at the neurosurgical outpatient clinic using the same grading scale after 3 and 12 months (Supplementary Table 1). In addition, we categorized symptom severity prior to treatment (surgery/conservative) and at 3 and 12 months follow-up (Supplementary Table 1).

The clinical routine in our department is to consider surgical treatment with extirpation of the pineal cyst in individuals with severe symptoms affecting occupational capacity, combined with MRI signs of a crowded pineal recess, as previously reported.^7^ Since there are no generally accepted criteria for surgery in these patients, the first author who performs the surgery (P.K.E.) is cautious in recommending surgery for non-hydrocephalic cysts in symptomatic patients. During the study period, absolute criteria for offering surgery were (i) severe symptoms heavily affecting quality of life and inability to work/schooling and (ii) anatomical evidence of crowding of the pineal recess, i.e. Grades 2–4 of central venous hypertension, according to grading of previously reported biomarkers of crowding within the pineal recess (Supplementary Table 2).^9^

#### Verification of absence of hydrocephalus

All MRI acquisitions were done using a 3 Tesla Philips Ingenia MRI scanner (Philips Medical systems, Best, The Netherlands). From T1 volume MRI, ventricular size was assessed by Evan’s index, and volume of the fourth, third and lateral ventricles, respectively, was estimated from FreeSurfer software (version 6.0; http://surfer.nmr.mgh.harvard.edu/).

### Imaging markers of a crowded pineal recess

**Figure 1:**
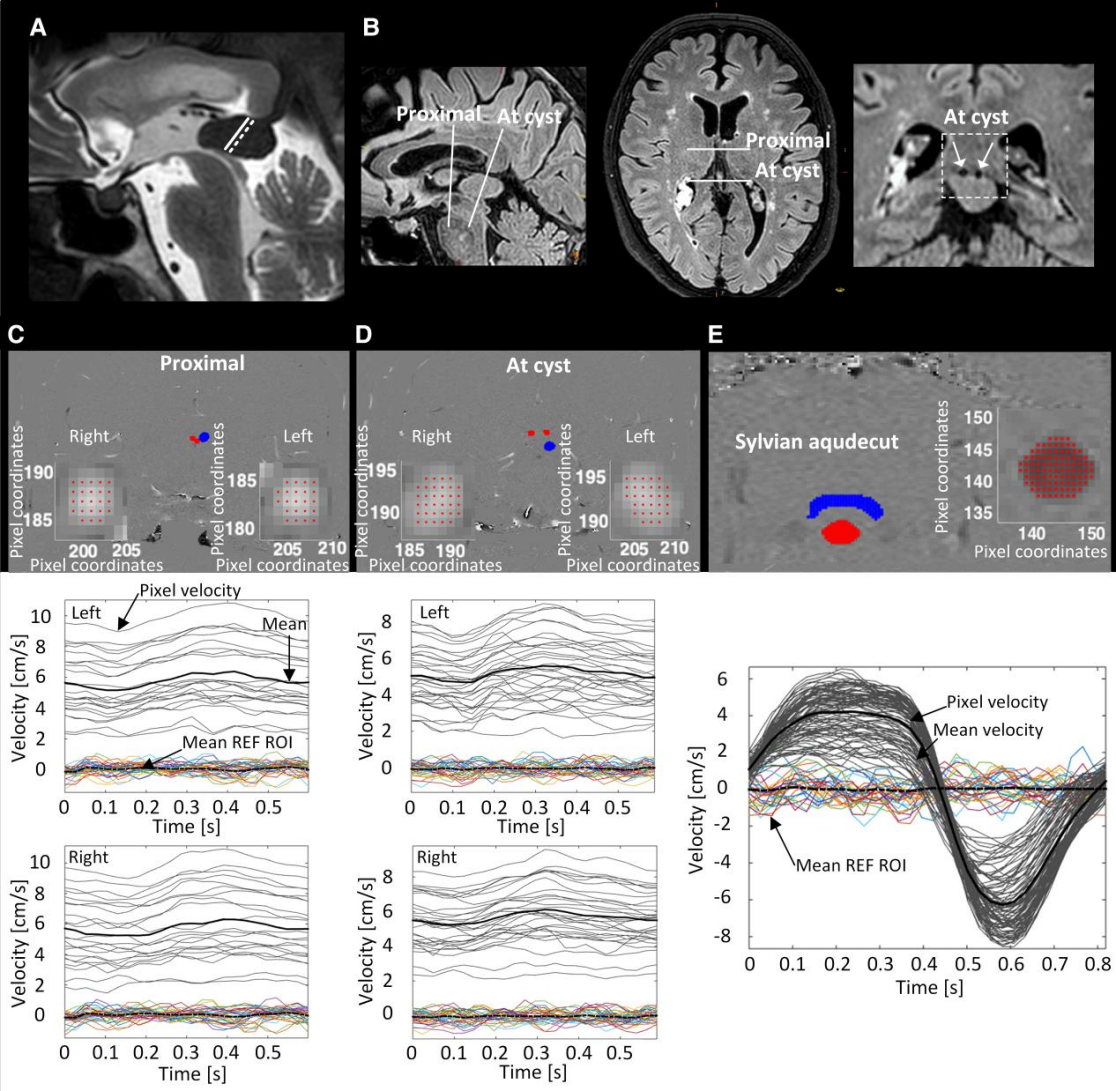
**Image-based biomarkers of physiological alterations associated with crowding of the pineal recess in symptomatic patients with non-hydrocephalic pineal cysts.** (**A**) The tectum-splenium-cyst ratio was defined from midsagittal MRI as the ratio between the pineal cyst diameter drawn at the shortest distance from the splenium of corpus callosum to the tectum divided by the length of the splenial-tectal distance drawn along the same line. (**B**) Blood flow in the internal cerebral veins was estimated proximal (upstream) to the cyst (proximal) and at the cyst (at cyst). (**C**) From phase-contrast MRI sequences, regions of interest (ROIs) were placed in the left and right internal cerebral veins (shown in red) proximal to the cyst (proximal). Reference ROIs (blue) were placed nearby. Zoom of the left vein at lower left and zoom of the right vein at lower right. Flow velocities were determined for every pixel within the ROI. Grey lines show the vein pixel velocities with black line referring to mean velocity of the vein. The coloured pixel velocities are from the reference ROI with black stippled line showing the mean velocity of the reference ROI. (**D**) Likewise, ROIs (red) were placed in the internal cerebral veins at the cyst (at cyst). Reference ROIs (blue) were placed nearby. Zoom of the left vein at lower left and zoom of the right vein at lower right. Flow velocities were determined for every pixel within the ROI. Grey lines show the vein pixel velocities with black line referring to mean velocity of the vein. The coloured pixel velocities are from the reference ROI with black stippled line showing the mean velocity of the reference ROI. (**E**) For estimation of net CSF flow in the Sylvian aqueduct, the ROI was placed within the Sylvian aqueduct (red) and the reference ROI (blue). Zoom of the Sylvian aqueduct to the right. Flow velocities were determined for every pixel within the ROI. Grey lines show the vein pixel velocities with black line referring to mean velocity of the vein. The coloured pixel velocities are from the reference ROI with black stippled line showing the mean velocity of the reference ROI.

We included two MRI markers of cyst-induced crowding of the pineal recess, namely the tectum-splenium-cyst ratio constituting an anatomical measure of crowding, and the thalamic apparent diffusion coefficient (ADC) ratio, serving as a marker of interstitial water content.^9^ Both biomarkers were previously found to differentiate mild from severe symptoms,^9^ intracranial pressure scores^10^ and outcome of surgery.^7^ These MRI biomarkers were defined from T1 volume midsagittal and axial diffusion-weighted MRI (1 mm thickness), as previously described,^9^ including the following:

- Tectum-splenium-cyst ratio. The cyst diameter was measured along the same line that defined the shortest distance between the tectum and the splenium, providing for the ratio between cyst diameter and the shortest distance from the splenium to the tectum in the same midsagittal image plane (Fig. 1A). We have selected this ratio as it may readily be applied to reproducibly assess to which degree the pineal cyst occupies the suprapineal recess/quadrigeminal cisternal space where the central veins of the brain traverse.
- Thalamic ADC ratio. The diffusion scans were oriented axially covering the whole brain, as previously reported.^9^ Evidence for increased interstitial water content was assessed by the apparent diffusion coefficient (ADC) ratio, defined as the ratio of ADC values between the thalamus and a standardized reference tissue (central white matter), to correct for variation in ADC between the MRI acquisitions. Since the absolute ADC values may depend on minor variations in magnetic field strengths and/or acquisition parameters at different time points of MRI scanning, we used the ADC ratio as a more robust score. A ROI located centrally in hemispheric white matter was used as a reference.^9^ To avoid partial volume effects, care was taken not to include neighbouring tissue elements or CSF. The underlying assumption is that ADC is dependent of interstitial water content.^14^

Two other measures of crowding of the pineal recess were included as they are commonly used to assess pineal cyst size:

- Anterior-posterior diameter of cyst. Pineal cyst size was determined as distance in millimetre from the maximum anterior–posterior diameter, using the midsagittal MRI.
- Tectum compression with aqueduct stenosis. From the midsagittal MRI, we also graded compression of the tectum as absent, moderate or severe. Aqueduct stenosis was dichotomized as absent, or present when there was a visually recognizable narrowing of the aqueduct lumen at sagittal T1. The combined tectum compression and aqueduct stenosis was dichotomized as no/moderate.

### Assessment of physiological variables possibly associated with crowding of the pineal recess

This study explored different physiological variables possibly accompanying crowding of the pineal recess: (i) alterations in blood flow velocity within the internal cerebral veins as they traverse immediately adjacent to the cyst, examined by phase-contrast MRI (PC-MRI); (ii) alterations in CSF flow velocity, examined by PC-MRI of the Sylvian aqueduct, and by intrathecal contrast-enhanced MRI as tracer enrichment within CSF spaces and (iii) alterations in intrathecal CSF tracer-mediated enhancement in extravascular brain tissue (as marker of glymphatic function) at MRI.

### Blood flow velocity in the internal cerebral veins and alterations in CSF flow

To measure change in blood flow velocity in the internal cerebral veins (Fig. 1B–D) and net flow rate in the Sylvian aqueduct (Fig. 1E), we used PC-MRI methodology as previously described.^15^

PC-MRI acquisitions were oriented perpendicular to the veins according to their delineation as structures with signal void from flow at sagittal FLAIR images. Velocities were measured proximal to (upstream) and at level with the cyst. Measurements from the left and right internal cerebral veins were performed separately. Three regions of interest (ROIs) were applied for each time series, two regions within the border of each of the two veins and one for reference velocities in stationary brain tissue close to the veins.

For CSF measurements in the Sylvian aqueduct, two ROIs were applied for each time series, one within the border of the aqueduct and one in the brain tissue adjacent to the cerebral aqueduct for reference velocities.

Data from PC-MRI were post-processed in MATLAB® (Mathworks, Natick, USA). The recorded velocities were converted by linear transformation from pixel values to centimetres per second by applying the velocity encoding. Reference velocities were used to capture and correct for any flow velocity baseline shift from zero, which may constitute a potential bias. Pixel velocities were adjusted for the baseline shift before further analysis. Aliased velocities, e.g. velocities that exceeded the velocity encoding, were corrected by a filter. The recorded velocities were averaged over the number of pixels in the region of interest to achieve a mean velocity.

Volumetric flow rate *Q*(ml/s) for each ROI was determined by computing the sum of each pixel velocity over one cycle multiplied with pixel size

$$Q(t)=\;\left(\overset{n}{\underset{i=1}{\sum }}\;v_{i\;}\right)\times dx\times dy$$

The stroke volume over one cycle was then calculated by discrete integration (trapezoidal method) of *Q* over time:

$$Stroke\;volume=\;\frac{dt}{2}\overset{n}{\underset{i=1}{\sum }}\;(Q(t_{i+1})+Q(t_{i\;}))$$

A more detailed description of the methodology has been presented previously.^15^

CSF volumes in the aqueduct over one cycle in cranial and caudal directions were calculated by integration of positive and negative volume fluxes over time. The CSF volumetric net flow rate during one cycle (ml/cycle) was determined by the sum of the positive and negative CSF flux. The daily CSF volumetric net flow rate, expressed in litre (L) over 24 h, was estimated by multiplying the CSF net flow volume over one cardiac cycle with the heart rate (HR) and then multiplied with 1440 (min/day). The HR was determined over the MRI scan time that was 6 min.

Alterations in CSF flow also were determined as percentage change in the T1 MRI signal, determined in different locations, i.e. in subarachnoid space at vertex, nearby the pineal cyst, in cisterna magna and in velum interpositum. Furthermore, percentage change in CSF tracer enrichment in the cerebral ventricles was determined. For details, see paragraph below, ‘Alterations in glymphatic function’.

To estimate change in venous flow at the cyst, we determined the percentage change in volumetric flow rate at the cyst versus proximal to the cyst (Fig. 1B–D). Flow measurements in veins may depend on angulation of section plane. Therefore, we manually controlled every instance, and excluded PC-MRI exams where veins deviated from the perpendicular axis of the imaging plane >10^°^.

### Alterations in glymphatic function

As a surrogate marker of glymphatic function in humans, we used intrathecal contrast enhanced MRI,^16,17^ where the MRI contrast agent gadobutrol (Gadovist, Bayer AG, GE) serves as CSF tracer. Identical MRI protocol settings (3D T1-weighted volume scans) were used before and at multiple time points up to 24–48 h after intrathecal CSF tracer injection: repetition time = ‘shortest’ (typically 5.1 ms), echo time = ‘shortest’ (typically 2.3 ms), flip angle = 8 degrees, field of view = 256 × 256 cm and matrix = 256 × 256 pixels (reconstructed 512 × 512). Hundred and eighty-four over-contiguous (overlapping) slices with 1 mm thickness were automatically reconstructed to 368 slices with 0.5 mm thickness. The duration of each image acquisition was 6 and 29 s. Slice orientation of image stacks was defined using an automated anatomy recognition protocol based on landmark detection in MRI data (SmartExam^tm^, Philips Medical Systems, Best, The Netherlands) for each time point to secure consistency and reproducibility of the MRI slice placement and orientation. Notably, all patients were examined in the same 3 T MRI scanner, using standardized MRI settings according to a defined protocol.

FreeSurfer software (version 6.0) (http://surfer.nmr.mgh.harvard.edu/) was applied for segmentation, parcellation and registration/alignment of the longitudinal data to estimate increase of T1 signal intensity.^18^ For every segmented area, we computed the median T1 signal unit for each time point, and divided the median signal unit against the signal unit of a reference ROI placed within the orbital fat tissue in axially reconstructed images from the same T1 volume scan. This signal unit ratio refers to the ‘normalized T1 signal units’ and adjusts for the baseline image grey scale changes between scans.

The intrathecal injection of gadobutrol was in each case done by an interventional neuroradiologist. For verification of correct position of syringe tip were used CSF backflow from the puncture needle verified correct position of the syringe tip in the subarachnoid space. Gadobutrol was given in a dose of 0.5 mmol (0.5 ml of 1.0 mmol/ml gadobutrol; Gadovist, Bayer Pharma AG, Berlin, Germany). The participants remained in bed until the last MRI acquisition at Day 1 (approximately at 4 p.m.), and were thereafter allowed free movement.

### Statistical analyses

Continuous data are presented as mean and standard deviation, standard error or 95% confidence interval; normal distribution of data was assessed in both groups. Categorical data were assessed using Pearson chi-square test, and continuous data by independent samples *t*-test. The repeated measurements on tracer enrichment within brain regions were assessed with linear mixed models, utilizing maximum likelihood estimation with a subject-specific random intercept and different residual errors at each time point or with unstructured residual errors depending on model fit. Mean, standard errors 95% confidence intervals and *P*-values were calculated from the linear mixed model for repeated measurements.

The statistical analysis was performed using SPSS version 26 (IBM Corporation, Armonk, NY) and Stata/SE 17.0 (StataCrop LLC, College Station, TX). Statistical significance was accepted at the 0.05 level (two-tailed).

### Data availability

The data presented in this work are available upon reasonable request.

## Results

### Patient material

Twenty-five symptomatic patients with non-hydrocephalic pineal cysts were included in the study (Table 1). Symptom profile is presented in Table 1. According to our previously reported grading scale,^9^ symptom severity was deemed minor in 4%, moderate in 20% and severe in 76% of patients. Due to the symptoms, 19/25 patients (76%) were unable to work or continue schooling. The MRI ventricular measures verified absence of hydrocephalus in all patients (Table 1).

**Table 1:** Patient material

***N***	25
***Gender (F/M)***	23/2
***Age (years)***	35.0 ± 10.6
***BMI (kg/m^2^)***	27.8 ± 5.4
***Symptoms prior to MRI***	
Headache (no-moderate/severe)	5/20
Nausea/vomiting (no/yes)	9/16
Dizziness (no/yes)	10/15
Visual disturbances (no/yes)	7/18
Episodic loss of consciousness (no/yes)	21/4
Lethargy/fatigue (no-moderate/severe)	4/21
Cognitive impairment (no-moderate/severe)	18/7
Transient neurologic deficits^a^ (no/yes)	10/15
***Grading of symptoms*** ^9^	
Minor (*n*, %)	1 (4%)
Moderate (*n*, %)	5 (20%)
Severe (*n*, %)	19 (76%)
***Occupational ability***	
Inability to work/schooling due to symptoms (*n*, %)	19 (76%)
***Subjective sleep quality***	
PSQI total score	10.2 ± 4.2
***MRI indices of hydrocephalus***	
Evans index	0.26 ± 0.03
Callosal angle (degrees)	120.4 ± 11.3
Volume 4th ventricle (ml)	1.4 ± 0.5
Volume 3rd ventricle (ml)	0. 8 ± 0.3
Volume lateral ventricles (ml)	15.5 ± 7.8
***MRI indices of crowded pineal recess***	
Tectum-splenium-cyst ratio	0.90 ± 0.14
Tectum-splenium-cyst ratio >0.9 (*n*, %)	15 (60%)
Thalamic ADC ratio	1.02 ± 0.03
Thalamic ADC ratio >1.01 (*n*, %)	12 (48%)
Anterior-posterior diameter of cyst (mm)	16.6 ± 4.5
Tectum compression + aqueduct stenosis (no/moderate)	16/9

a Transient neurologic deficits include tremor, paraesthesia, impaired coordination and motor weakness. Categorical data presented as numbers (ranges in parentheses); continuous data presented as mean ± standard deviation. ADC, apparent diffusion coefficient; BMI, body mass index; MRI, magnetic resonance imaging; PSQI, Pittsburgh sleep quality index.

Some degree of crowding in the pineal recess was seen in all subjects. According to our previously described criteria for crowding of the pineal recess,^9^ crowding was present in 23/25 (92%) patients, which corresponds to our previously reported pineal cyst grade of 2–4 (Supplementary Table 2).^9^

The MRI-based physiological measures explored in this study are presented in Table 2.

**Table 2:** Physiological measures of blood flow in the internal cerebral veins and CSF flow in the cerebral aqueduct, and enrichment of a CSF tracer in CSF spaces and the brain

***Flow changes in the internal cerebral veins before and at cyst (n = 18)***	
Change in maximum flow velocity at cyst, left (%)	7.8 ± 21.6
Change in maximum flow velocity at cyst, right (%)	7.2 ± 16.7
Change in mean flow velocity at cyst, left (%)	9.1 ± 20.7
Change in mean flow velocity at cyst, right (%)	8.5 ± 18.2
Change in stroke volume at cyst, left (%)	20.5 ± 22.1
Change in stroke volume at cyst, right (%)	18.4 ± 24.0
***CSF flow changes in the cerebral aqueduct (n = 20)***	
*Antegrade-directed net flow*	
*N* (%)	15 (75%)
Volume (ml/cycle)	0.003 ± 0.002
Estimated volume (L/24 h)	0.277 ± 0.168
*Retrograde-directed net flow*	
N (%)	5 (25%)
Volume (ml/cycle)	0.005 ± 0.005
Estimated volume (L/24 h)	0.447 ± 0.533
***Ventricular reflux grading (n = 25)***	
Grades 0-1/2-4	15/10
***Tracer enrichment in CSF (n = 25)***	
Cisterna magna (6 h; %)	4540 ± 1269
Cisterna magna (24 h; %)	1179 ± 885
Nearby pineal cyst (6 h; %)	2419 ± 1043
Nearby pineal cyst (24 h; %)	746 ± 777
Vertex (6 h; %)	2194 ± 2665
Vertex (24 h; %)	2530 ± 2102
4th ventricle (6 h; %)	883 ± 697
4th ventricle (24 h; %)	151 ± 119
3rd ventricle (6 h; %)	367 ± 456
3rd ventricle (24 h; %)	64 ± 68
Lateral ventricles (6 h; %)	241 ± 327
Lateral ventricles (24 h; %)	34 ± 35
***Tracer enrichment in the brain (n = 25)***	
Cerebral cortex (6 h; %)	111 ± 46
Cerebral cortex (24 h; %)	95 ± 46
Subcortical white matter (6 h; %)	11 ± 7
Subcortical white matter (24 h; %)	34 ± 17
Thalamus (6 h; %)	12 ± 6
Thalamus (24 h; %)	18 ± 9

Categorical data presented as numbers (ranges in parentheses); continuous data presented as mean ± standard deviation.

### Blood flow in deep cerebral veins

**Figure 2:**
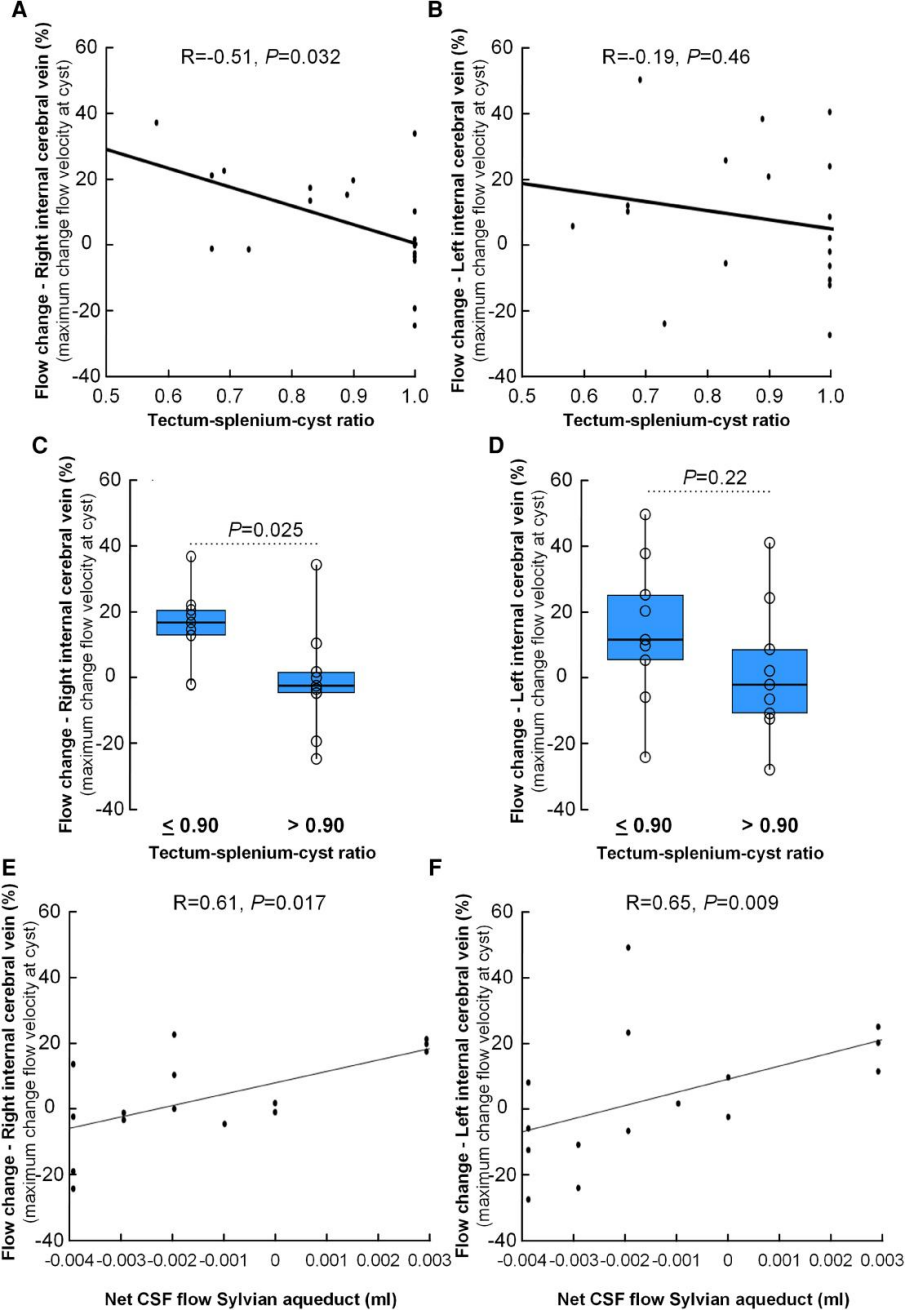
**Flow changes in the internal cerebral veins associated with cyst-induced crowding of the pineal recess.** Association between the tectum-splenium-cyst ratio and blood flow change in **A** the left internal cerebral vein and **B** the right internal cerebral vein (measured as percentage change in maximum flow velocity at the cyst) (*n* = 18). For each plot, the fit line and Pearson correlation coefficient (R) with significant *P*-value are shown. For the tectum-splenium-cyst ratio thresholds of either ≤0.90 or >0.90, the differences in maximum blood flow velocity change in **C** the left internal cerebral vein and **D** the right internal cerebral vein are shown (*n* = 19) as box plots with individual data points. *P*-value determined by independent samples *t*-test. Net CSF flow in the Sylvian aqueduct (ml) correlated with blood flow change in **E** the left internal cerebral vein and **F** the right internal cerebral vein (measured as percentage change in maximum flow velocity at the cyst) (*n* = 15). For each plot, the fit line and Spearman’s correlation coefficient (R) with significant *P*-value are shown.

Supplementary Table 3 presents the blood flow estimates in the internal cerebral vein, determined proximal (upstream) to the cyst and at the level of the cyst (see Fig. 1B–D), including various measures of change in blood flow at the cyst relative to the proximal level. The various blood flow estimates tended to increase at the cyst (Supplementary Table 3). Video 1 shows a 3D visualization of flow in the internal cerebral veins proximal to the pineal cyst, while Video 2 shows a 3D visualization of flow in the internal cerebral veins at the pineal cyst. Notably, the percentage change was significantly lower with increasing crowding of the pineal recess (Fig. 2). Hence, there was a significant negative correlation between the tectum-splenium-cyst ratio and percentage increase in maximum flow velocity at the cyst within the right internal cerebral vein (Fig. 2A). This finding suggests that with increasing crowding, the increase in maximum blood flow velocity at the cyst became diminished. This effect was not significant for the left internal cerebral vein (Fig. 2B). Moreover, the percentage change in maximum blood flow velocity at the cyst for the right internal cerebral vein was significantly different for the tectum-splenium-cyst ratio ≤0.90/>0.90 (Fig. 2C), though not for the left internal cerebral vein (Fig. 2D). Similar results were found when assessing percentage change in mean blood flow velocity (data not shown). The change in internal cerebral vein stroke volume at the cyst did not correlate with the tectum-splenium-cyst ratio (data not shown).

Furthermore, there was a significant positive correlation between net CSF flow per cycle in the Sylvian aqueduct and percentage change in maximum blood flow velocity at the cyst, both at the right (Fig. 2E) and left (Fig. 2F) internal cerebral veins. The same was observed when utilizing the mean blood flow velocity of the internal cerebral veins (data not shown). These findings indicate an association between the venous blood flow velocity change at the cyst and net CSF flow in the Sylvian aqueduct.

### Distribution of CSF tracer in CSF spaces

Some observations suggest altered CSF flow in patients with cyst-induced crowding of the pineal recess. Thus, PC-MRI showed retrograde net CSF flow in the Sylvian aqueduct in 5/20 (25%) patients, and intrathecal contrast-enhanced MRI demonstrated reflux Grades 2–4 in 10/25 (40%) patients (Table 2). Furthermore, ventricular reflux Grades 2–4 were seen in 3/5 (60%) patients with net retrograde CSF flow at PC-MRI while ventricular reflux Grades 2–4 were seen in 6/15 (40%) subjects with PC-MRI evidence of anterograde net CSF flow. Independent of MRI methodology, 12/25 patients (48%) showed evidence of retrograde CSF towards ventricles, indicative of altered CSF flow, which may affect transport of substances to/from CNS even in the absence of hydrocephalus.

With regard to MRI markers of crowding within the pineal recess, the tectum-splenium-cyst ratio was not correlated with CSF tracer enrichment within the CSF spaces (cisterna magna, nearby cyst, vertex or within the fourth, third or lateral ventricles) (data not shown).

### Glymphatic function

**Figure 3:**
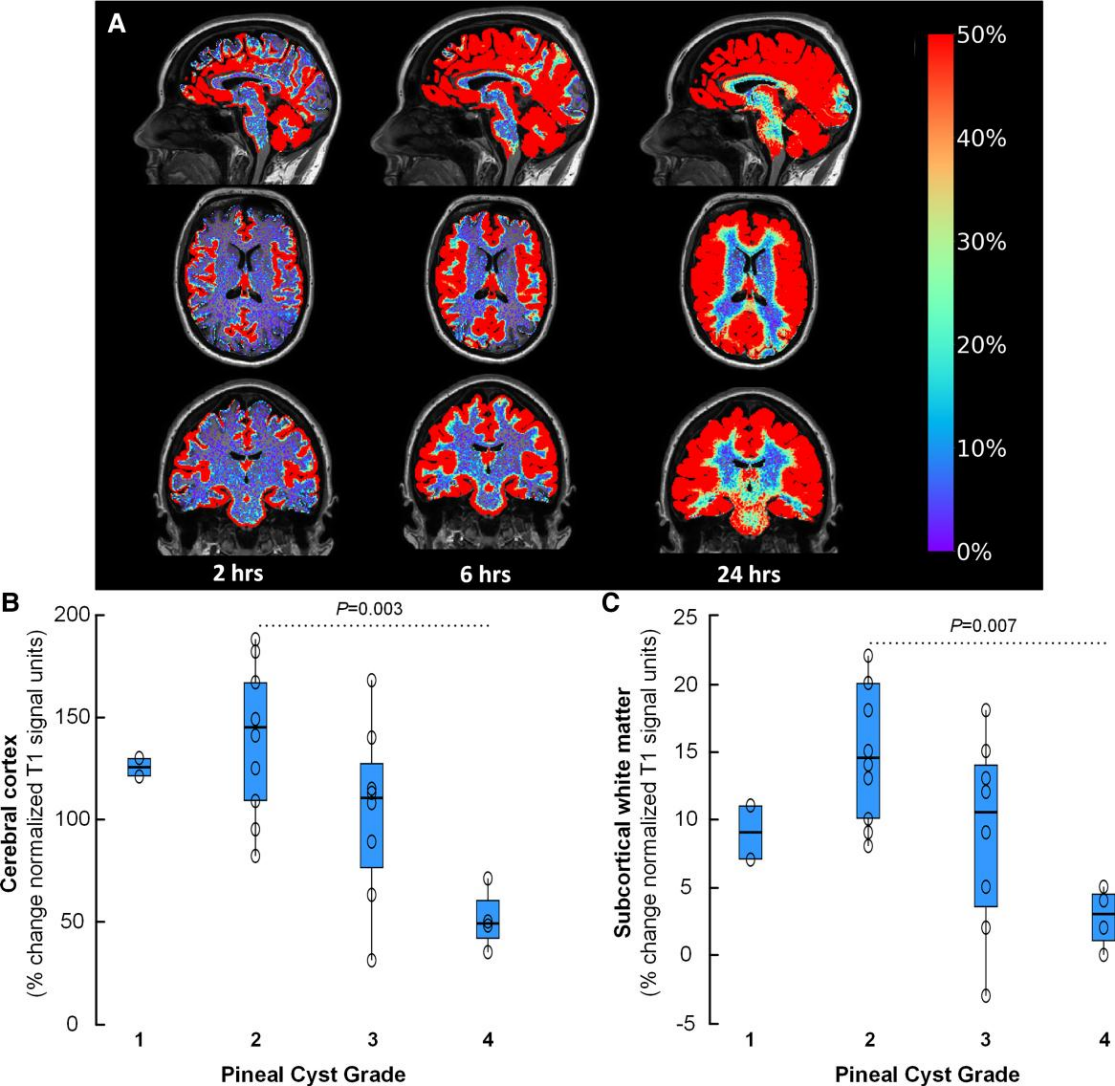
**Glymphatic enrichment in the cerebral cortex and subcortical white matter versus crowding in pineal recess assessed according to the PC grading scale.**^9^ (**A**) Glymphatic transport in patients with pineal cysts was examined by intrathecal contrast-enhanced MRI, showing tracer enrichment as change in T1 signal intensity over time (2, 6 and 24 h), here illustrated by percentage increase in normalized T1 signal unit ratios, as indicated on the bar to the right. The percentage change in tracer enrichment (normalized T1 signal) from baseline to 6 h after intrathecal tracer (gadobutrol) in **B** the cerebral cortex and **C** the subcortical white matter is compared for patient categories with PC Grading 1 (*n* = 2), 2 (*n* = 11), 3 (*n* = 8) and 4 (*n* = 4). Data presented as box plots with individual data points. There were overall differences between groups in both the cerebral cortex (**A**; *P* = 0.004) and subcortical white matter (**B**; *P* = 0.009), and significant differences between Groups 2 and 4 for both locations (ANOVA with Bonferroni corrected *post hoc* testing).

Glymphatic function was estimated by FreeSurfer-based determination of percentage change in normalized T1 signal unit ratio (Fig. 3A). Most interestingly, there was significantly reduced tracer enrichment within the cerebral cortex (Fig. 3B) and subcortical white matter (Fig. 3C) with increasing pineal cyst (PC) grade, which incorporates both the tectum-splenium-cyst ratio and thalamic ADC ratio (Supplementary Table 2),^9^ and serving as a measure of degree of crowding within the pineal recess. Hence, these observations indicate brain-wide effects of increasing crowding of the pineal recess, with impaired CSF-mediated molecular tracer enrichment from the brain along extravascular pathways, here represented by the cerebral cortex and subcortical white matter.

**Figure 4:**
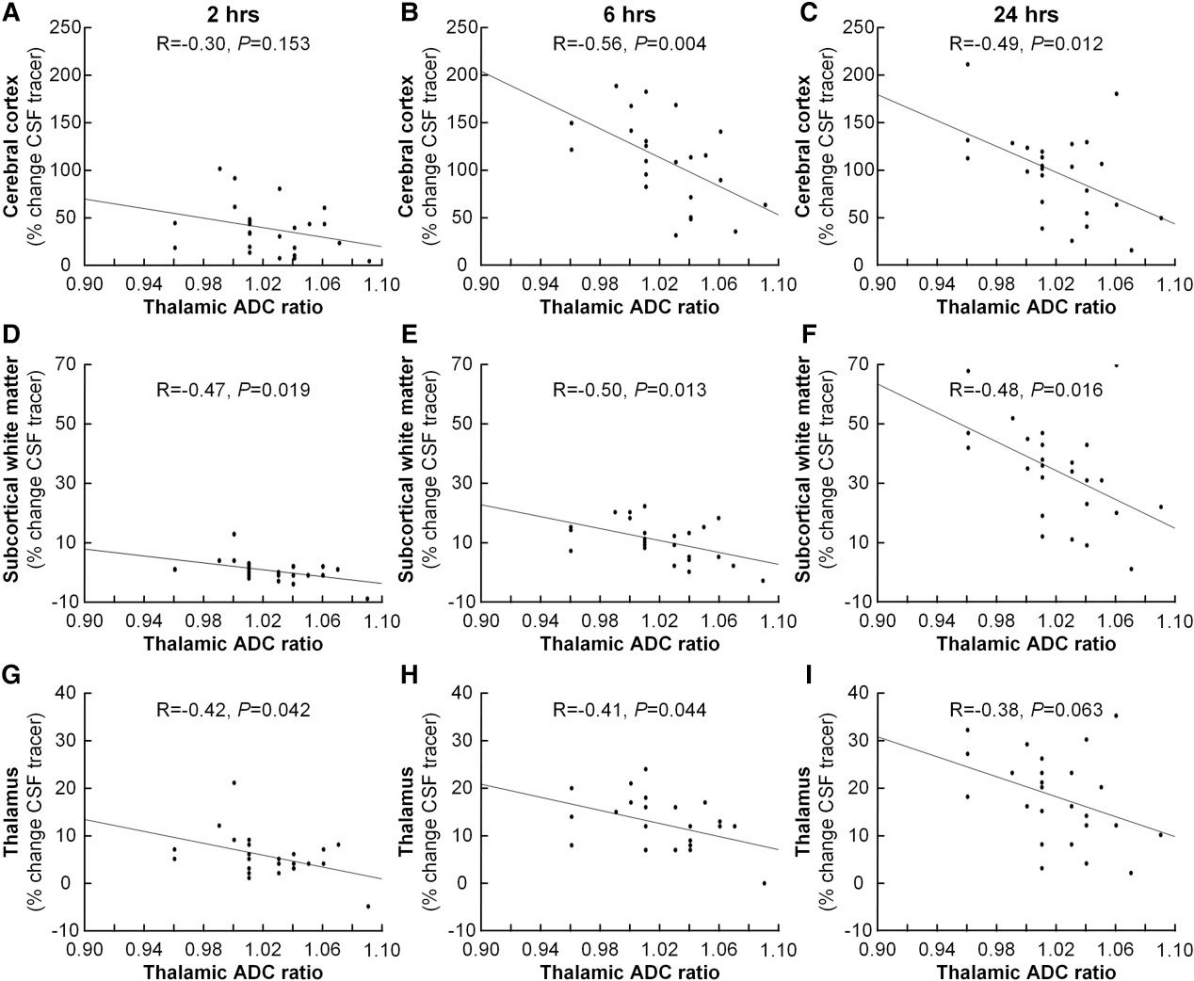
**Association between thalamic ADC ratio and tracer enrichment in the cerebral cortex, subcortical white matter and thalamus.** The association between thalamic ADC ratio and tracer enrichment (percentage change in normalized T1 signal) in the cerebral cortex after **A** 2 h (*n* = 24), **B** 6 h (*n* = 24) and **C** 24 h (*n* = 25), between thalamic ADC ratio and tracer enrichment in the subcortical white matter after **D** 2 h (*n* = 24), **E** 6 h (*n* = 24) and **F** 24 h (*n* = 25) and between thalamic ADC ratio and tracer enrichment in the thalamus after **G** 2 h (*n* = 24), **H** 6 h (*n* = 24) and **I** 24 h (*n* = 25). The tentative interpretation of these significant negative correlations at different time points is that increasing ADC ratio indicates increasing interstitial water content causing increasing interstitial pressure that in turn impairs glymphatic tracer enrichment. For each plot, the fit line and Pearson correlation coefficient (R) with significant *P*-value are shown.

Furthermore, looking at the individual components of the PC grade, we found that the thalamic ADC ratio correlated negatively with enrichment of CSF tracer in the cerebral cortex (Fig. 4A–C), subcortical white matter (Fig. 4D–F) and thalamus (Fig. 4G–I), while the tectum-splenium-cyst ratio was not correlated with tracer enrichment within the brain (data not shown). Assuming that increased ADC ratio is indicative of higher interstitial water content, we interpret these findings as being indicative of higher interstitial water content resulting in increased interstitial pressure and reduced glymphatic enrichment. In line with this assumption, a thalamic ADC ratio >1.01 was accompanied with significantly reduced tracer enrichment in the cerebral cortex at 6 h (137 ± 33% versus 86 ± 44%; *P* = 0.004), subcortical white matter at 2 h (1 ± 4% versus −2 ± 3%; *P* = 0.023) and 6 h (14 ± 5% versus 7 ± 7%; *P* = 0.007) as well as in the thalamus at 6 h (15 ± 6% versus 10 ± 5%; *P* = 0.029).

An interesting observation was that tracer enrichment in the thalamus correlated significantly with tracer enrichment in subarachnoid spaces at vertex, cisterna magna and nearby pineal cyst, while it did not correlate with tracer enrichment within the third or lateral ventricles, despite its close anatomical relationship (Supplementary Table 4). This indicates that tracer enrichment may be from the brain surface facing the subarachnoid compartment and not through the ependymal lining of the ventricles.

### Outcome after surgical cyst removal

Twenty-three of 25 patients had tectum-splenium-cyst ratio >0.90 and/or thalamic ADC ratio >1.01, corresponding to our previously reported PC Grades 2–4.^9^ Among these 23 patients, 11 underwent surgery with extirpation of the pineal cyst (Supplementary Table 5). Comparisons of patients treated surgically or conservatively showed that symptom severity prior to treatment was significantly worse in the surgery group (Supplementary Table 5). The indication for recommending surgery relies on the degree of symptoms and impact on occupational activity and schooling, and the MRI signs crowding in the pineal recess (i.e. PC Grades 2–4). Probably related to the fact that 23/25 of the present cases presented with PC Grades 2–4, the MRI signs of crowding were not significantly different between surgically and conservatively managed patients (Supplementary Table 6). Accordingly, only symptom severity differentiated the surgery/conservative groups.

At follow-up (observation period, mean ± SD; 1.9 ± 1.2 years), 10/11 (91%) patients reported marked improvement of symptoms and 1/11 (9%) some improvement. In comparison, among the 14 individuals managed conservatively, at follow-up (observation period, mean ± SD; 3.1 ± 1.6 years), 1/14 (7.1%) reported marked clinical improvement, 2/14 (14.3%) some improvement while 10/14 (71.4%) were clinically unchanged and 1/14 (7.1%) reported worsening of symptoms. The differences in symptomatic outcome between patients managed surgically or conservatively were significant (*P* < 0.001, Pearson chi-square test; Supplementary Table 7). Accordingly, removal of the pineal cyst and thereby relief of the crowding of the pineal recess was accompanied with marked improvement of symptoms. Concerning occupational capacity, in the surgery group, 8 of the 11 patients (73%) had regained ability to work/schooling, while in the conservative group, 5/14 (36%) had regained ability to work/schooling (*P* < 0.093, Pearson chi-square test).

**Figure 5:**
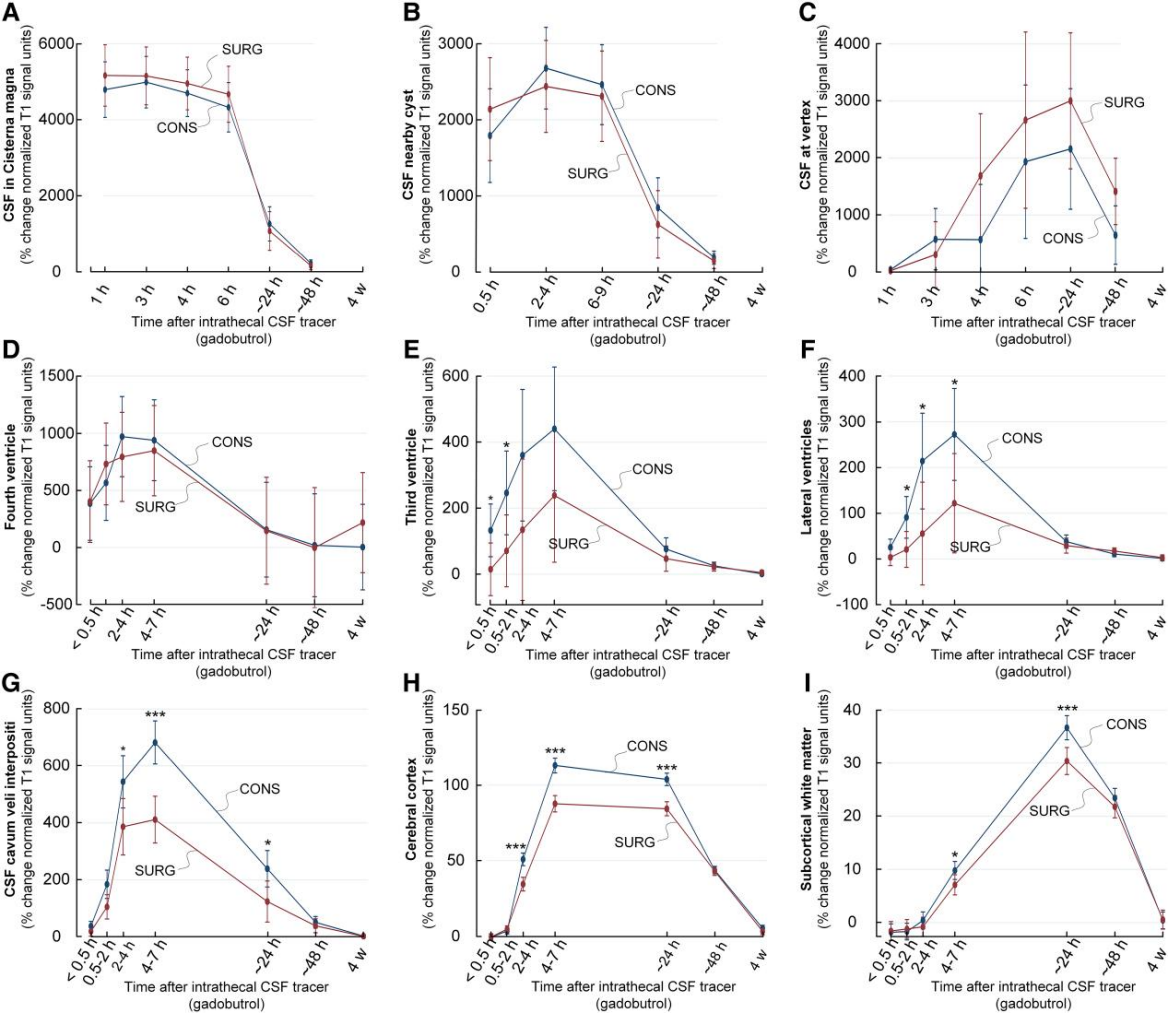
**Enrichment of tracer in intracranial CSF spaces and glymphatic enrichment over time in the patients managed surgically (SURG, *n* = 11) or conservatively (CONS, *n* = 14) for their pineal cysts.** We determined percentage change in tracer enrichment in CSF spaces after its lumbar intrathecal injection (see Fig. 1C), including the CSF spaces of the **A** cisterna magna, **B** subarachnoid space nearby the pineal cyst, **C** subarachnoid space at vertex, **D** fourth ventricle, **E** third ventricle, **F** lateral ventricles, **G** CSF of velum interpositum, **H** cerebral cortex and **I** subcortical white matter. Enrichment of CSF tracer is presented as percentage change in normalized T1 signal unit ratio with trend plots shown as mean ± 95% confidence interval. Significant differences between groups are indicated by *P < 0.05, **P < 0.01, ***P < 0.001 (linear mixed models).

Comparing enrichment of CSF between the surgically and conservatively managed patients demonstrated no differences in CSF tracer in subarachnoid spaces in cisterna magna (Fig. 5A), nearby pineal cyst (Fig. 5B) or at vertex (Fig. 5C), and no difference in enrichment within the fourth ventricle (Fig. 5D). On the other hand, CSF tracer enrichment was lower in the third ventricle (Fig. 5E), lateral ventricles (Fig. 5F) and velum interpositum (Fig. 5G) of the surgery group. Notably, glymphatic enrichment was reduced in the surgery group within cerebral cortex (Fig. 5H) and subcortical white matter (Fig. 5I). This was further evident for a wide range of brain regions (Supplementary Table 8). Therefore, in the surgically treated patients, who differed by severity of symptoms, glymphatic enrichment was reduced.

## Discussion

The present study provides evidence that in symptomatic individuals with non-hydrocephalic pineal cyst, crowding of the pineal recess is accompanied with physiological alterations in blood flow of the internal cerebral veins and alterations in CSF flow, as well as brain-wide changes in glymphatic function. With increasing tectum-splenium-cyst ratio, indicative of more pronounced crowding, the increase in venous blood flow velocity at the cyst was reduced, which was associated with increased retrograde CSF net flow in the Sylvian aqueduct. Remarkably, with increasing crowding (i.e. higher PC grade), glymphatic tracer enrichment became impaired in the cerebral cortex as well as in the subcortical white matter. Moreover, with increasing thalamic ADC ratio, tracer enrichment was reduced in the thalamus, indicative of impaired glymphatic enrichment. Reduced tracer enrichment in the cerebral cortex, subcortical white matter and thalamus was also demonstrated in the patients with severe symptoms undergoing surgery and with symptomatic improvement after surgery.

It should be kept in mind that the present cohort of symptomatic individuals with non-hydrocephalic pineal cysts represents a minor subset of individuals with pineal cysts as the vast majority of pineal cysts occur in patients with symptoms considered unrelated to cysts and is thereby categorized as incidental MRI findings.^2,19,20^ The majority of individuals present with small cysts while the average anterior–posterior cyst diameter in the present cases were 16.6 ± 4.5 mm. Therefore, the present results relate to larger cysts accompanied with symptoms, though without hydrocephalus.

The common way of assessing crowding of the pineal recess is by measuring the anterior–posterior diameter of the cyst in the midsagittal plane or by categorical assessment of tectal compression and stenosis of the Sylvian aqueduct.^5^ However, in a previous study, we found no association between these measures and symptom severity in symptomatic individuals with non-hydrocephalic pineal cysts.^9^ On the other hand, we previously reported that two imaging markers of a crowded pineal recess, namely the tectum-splenium-cyst ratio and the thalamic ADC ratio (that provided for the PC grade; Supplementary Table 2), significantly associated with symptom severity,^9^ intracranial pulsatile pressure^10^ and outcome of surgery for pineal cysts.^7^ Therefore, we found a good rational behind applying these imaging markers of pineal recess crowding.

We introduced the tectum-splenium-cyst ratio as a measure of the degree of venous compromise at the cyst since pineal cysts may dislocate the internal cerebral veins towards the splenium of corpus callosum above, or laterally, as they traverse the superior or lateral convexity of the cyst.^9,10^ The present observations from PC-MRI disclosed in cysts with less crowding greater change in internal cerebral vein blood velocity at the cyst as compared with proximal to the cyst (Fig. 1B–D). More pronounced crowding (i.e. higher tectum-splenium-cyst ratio), on the other hand, was accompanied with less increase in venous blood flow velocity at the cyst. We have no good explanation why the change in blood flow velocity is reduced with increasing degree of crowding, but consider it to be of significance that there exists an association between degree of crowing and degree of blood flow velocity change at cyst level. Currently, we have limited understanding of venous flow changes in humans. PC-MRI was previously used to assess flow velocity in cerebral veins (superior sagittal sinus, straight sinus, and transverse sinus), and showed a high degree of inter-individual variation, variable side-dominance in paired veins and even a significant contribution of accessory epidural outflow in addition to the jugular vein outflow.^21^ Variable side-dominance may perhaps explain why we found significant results for the right internal cerebral vein only. In the present material, estimates of venous blood flow velocity in the internal cerebral vein tended to be higher at the level of the cyst, as compared to proximal to the cyst (Supplementary Table 3). Due to the high degree of variation, each patient was his/her control, and blood flow velocity change at cyst relative to proximal (upstream) was determined. Our interpretation is that cyst-induced crowding may alter blood flow velocity by changing the blood vessel configuration, though we lack proof for this assumption. Further studies are needed to confirm this.

We here also provide evidence for an association between change in venous blood flow at the cyst and the net CSF flow in the Sylvian aqueduct. In addition, the findings of retrograde net CSF flow in the Sylvian aqueduct in 25%, ventricular reflux of CSF tracer Grades 2–4 in 40% of patients and evidence of ventricular reflux independent of method in 48% of patients point at CSF flow alterations even though hydrocephalus was verified to be absent. Traditionally, symptoms in subjects with pineal cysts have been attributed to hydrocephalus, and clinical improvement following resection of symptomatic non-hydrocephalic cysts has been explained by intermittent blockade of CSF flow.^5,22^ The present data provide further support to an assumption that CSF flow may be altered even though it is not accompanied with ventriculomegaly indicative of hydrocephalus. Furthermore, adding to the evidence of altered CSF flow, we previously reported alterations in CSF to blood clearance (i.e. increased lag time) in symptomatic patients with non-hydrocephalic pineal cysts.^23^ Redistribution of CSF could explain a prolonged lag time in these patients, as ventricular reflux will supposedly delay tracer arrival at potential clearance routes near the CNS outer borders.

A most intriguing observation was the association between crowding of the pineal recess and glymphatic enrichment. In particular, higher PC grade, indicative of higher degree of crowding, was accompanied with reduced glymphatic enrichment (Fig. 3), and increased thalamic ADC ratio was accompanied with reduced glymphatic enrichment at various time points in the cerebral cortex, subcortical white matter and thalamus (Fig. 4). We previously reported that patients with pineal cyst Grades 2–4 experienced marked improvement in symptoms following surgical removal of the cyst.^7^ Individuals with Grades 2–4 also had increased risk of developing severe symptoms.^9^ Furthermore, the individuals who improved after surgical cyst removal showed impaired glymphatic enrichment in a wide range of brain regions (Supplementary Table 8). We have selected the thalamic ADC ratio^9^ as imaging marker of pineal recess crowding based on an assumption that ADC expresses interstitial water content.^14^ It may be speculated why increased ADC ratio in the thalamus should be accompanied with brain-wide impairment of glymphatic tracer enrichment, including the cerebral cortex and subcortical white matter, and not be restricted to regions nearby the pineal recess. This question cannot be fully answered here, but the data point towards mechanisms affecting the entire brain, where one such overriding mechanism may be hormonally mediated from the pineal gland itself. Nevertheless, according to our hypothesis (Supplementary Fig. 2), we suggest that altered venous flow in the deep cerebral veins is accompanied with increased interstitial thalamic water content and thereby higher interstitial fluid pressure, which in its turn impair glymphatic enrichment locally. Obviously, further studies are needed to clarify whether this assumption is correct.

Notably, the impaired glymphatic function seen in patients with higher PC grade was not preceded by lower tracer enrichment in CSF spaces at vertex. Rather, the impaired tracer enrichment compared with the experimental observations of markedly reduced glymphatic function during the awake state,^24^ which led the authors to conclude that that the glymphatic system is primarily active during sleep. Melatonin, the hormone produced by the pineal gland, has a well-established role in circadian control and sleep disturbance.^12^ It may be hypothesized that a large pineal cyst is secondary to retention of cyst content, thereby preventing hormonal release into CSF at initiation of sleep. In the present cohort, 21/24 (87.5%) patients had impaired sleep quality, i.e. PSQI total score >6. Only 3/24 (12.5%) patients had no subjectively impaired sleep quality, i.e. PSQI total score ≤5. On the other hand, Májovský *et al*.^25^ reported that individuals with a pineal cyst had preserved diurnal variation in serum melatonin concentrations while pineal cyst removal caused loss of endogenous melatonin production. In addition to sleep, melatonin has several other modulatory effects on various functions of the central nervous system.^26^ Of particular relevance, melatonin has been proposed to have a role in the headache, which is a major symptom in these patients.^27^ Currently, there is limited information about the normal transport route of melatonin via the CSF pathways, but most likely this transport is altered to a variable degree in subjects with pineal cysts. In this regard, the present observations of altered glymphatic transport could be particularly relevant, as it might affect transport of melatonin within the brain.

In this context, it may be of note that in animals, the pineal recess seems to be a main CSF influx site to the glymphatic circulation.^28^ Using MRI contrast agents to assess glymphatic flow in rats,^28^ entrance of CSF tracer into the paravascular space was by fast influx kinetics at two key nodes: the pituitary- and the pineal gland recesses. We found that tracer enrichment in cavum velum interpositum was significantly reduced in the surgery group (Fig. 5G), though not in the subarachnoid space posterior to the cyst. The altered CSF flow related to pineal cysts could be of importance for the normal transport and distribution of trophic substances such as melatonin.

Intrathecal contrast-enhanced MRI may presently be considered as gold standard for *in vivo* human assessment of glymphatic function.^16^ An underlying assumption is that the tracer (gadobutrol) reflects the extravascular transport of other water-soluble substances, even though the resolution of MRI (1 mm) does not allow for definite conclusion of tracer movement along paravascular (glymphatic) versus interstitial pathways. Interestingly, tracer studies in the gyrencephalic pig brain verified predominant paravascular tracer transport.^29^ The glymphatic system is a brain-wide paravascular clearance system for fluid and solutes dependent on CSF influx along arteries.^11,30^ It should be noted that the glymphatic system is still extensively debated as the evidence for several aspects of the concept remains weak.^31^ With the notation *glymphatic* enrichment, we essentially refer to CSF-mediated molecular enhancement in the brain along extravascular pathways. In this regard, impaired glymphatic function may both hamper the supply of nutrients and trophic factors necessary for normal brain function and the removal of waste substances from brain metabolism.^11^ We suggest the present observations of impaired glymphatic enrichment in individuals with high-grade pineal cysts may be suggestive of abnormal transport of melatonin that in turn may affect sleep function. The glymphatic system is primarily active during sleep.^24^ Therefore, it is of note that the present cases presented with marked subjective sleep disturbance.

Some limitations with the study should be noted. Flow measurements from PC-MRI have weaknesses, particularly when small amounts are measured, as we previously have commented on.^15^ Not least, this limitation is relevant for estimation of net flow changes within the Sylvian aqueduct. On the other hand, our approach has a methodological advantage since the measurements were made pixel-wise within the ROI, thereby increasing accuracy. Furthermore, since flow measurements in the veins depend on angulation of section plane, this was manually controlled in every instance, and we excluded PC-MRI exams where the veins deviated from the perpendicular axis of the imaging plane >10^°^. Nevertheless, some inaccuracy due to angulation differences cannot be excluded, even though this should be randomly distributed. Another limitation is that the present cohort was rather uniform (23/25 patients with pineal cyst Grade 2–4) with severe symptoms and marked indices of pineal recess crowding in the majority. A wider distribution of symptom severity and MRI markers of pineal recess crowding would have been useful for the analysis. Further, the intrathecal contrast-enhanced MRI method to estimate glymphatic function is limited by the resolution of MRI (1 mm), and low time resolution (MRI after 2, 6 and 24 h), On the other hand, despite limitations with the imaging methodology, this is the first study attempting to decipher mechanisms behind symptoms in patients with cyst-induced crowding of the pineal recess.

In conclusion, we interpret these present observations to support the hypothesis that cyst-induced crowding of the pineal recess without hydrocephalus may alter blood flow of the internal cerebral veins and CSF flow and even cause brain-wide impairment of glymphatic transport. The latter was seen in a subset of patients who underwent surgical removal of the cyst and who experienced marked symptom relief thereafter.

## Supplementary Material

fcad078_Supplementary_Data

## Abbreviations

ADC =: apparent diffusion coefficient
PC-MRI =: phase-contrast MRI
ROI =: region of interest
