# Supplementary material for: Physiological alterations of pineal recess crowding in symptomatic non-hydrocephalic pineal cysts
Source: Brain Commun. 2023 Mar 17;5(2):fcad078. doi: 10.1093/braincomms/fcad078 (PMC10371044; doi:10.1093/braincomms/fcad078)
Supplement: fcad078_Supplementary_Data [file fcad078_Supplementary_Data.pdf]

Supplementary Material

Physiological alterations of pineal recess crowding in symptomatic non-hydrocephalic pineal cysts

Per Kristian Eide[1,2*], Erika Kristina Lindstrøm,[3] Are Hugo Pripp[4,5], Lars Magnus Valnes[2] Geir Ringstad[6,7]

[1]Institute of Clinical Medicine, Faculty of Medicine, University of Oslo, Oslo, Norway.
[2]Department of Neurosurgery, Oslo University Hospital – Rikshospitalet, Oslo, Norway.
[3]Institute for cancer genetics and informatics, Oslo University hospital
[4]Oslo Centre of Biostatistics and Epidemiology, Research Support Services, Oslo University Hospital, Oslo, Norway.
[5]Faculty of Health Sciences, Oslo Metropolitan University, Oslo, Norway.
[6]Department of Radiology, Oslo University Hospital- Rikshospitalet, Oslo, Norway.
[7]Department of Geriatrics and Internal medicine, Sorlandet Hospital, Arendal, Norway.

Corresponding author:

Professor Per Kristian Eide, MD PhD
Department of Neurosurgery
Oslo University Hospital - Rikshospitalet
Pb 4950 Nydalen,
Phone: +47 91649419
Fax: +47-23074310
N-0424 Oslo, Norway
p.k.eide@medisin.uio.no

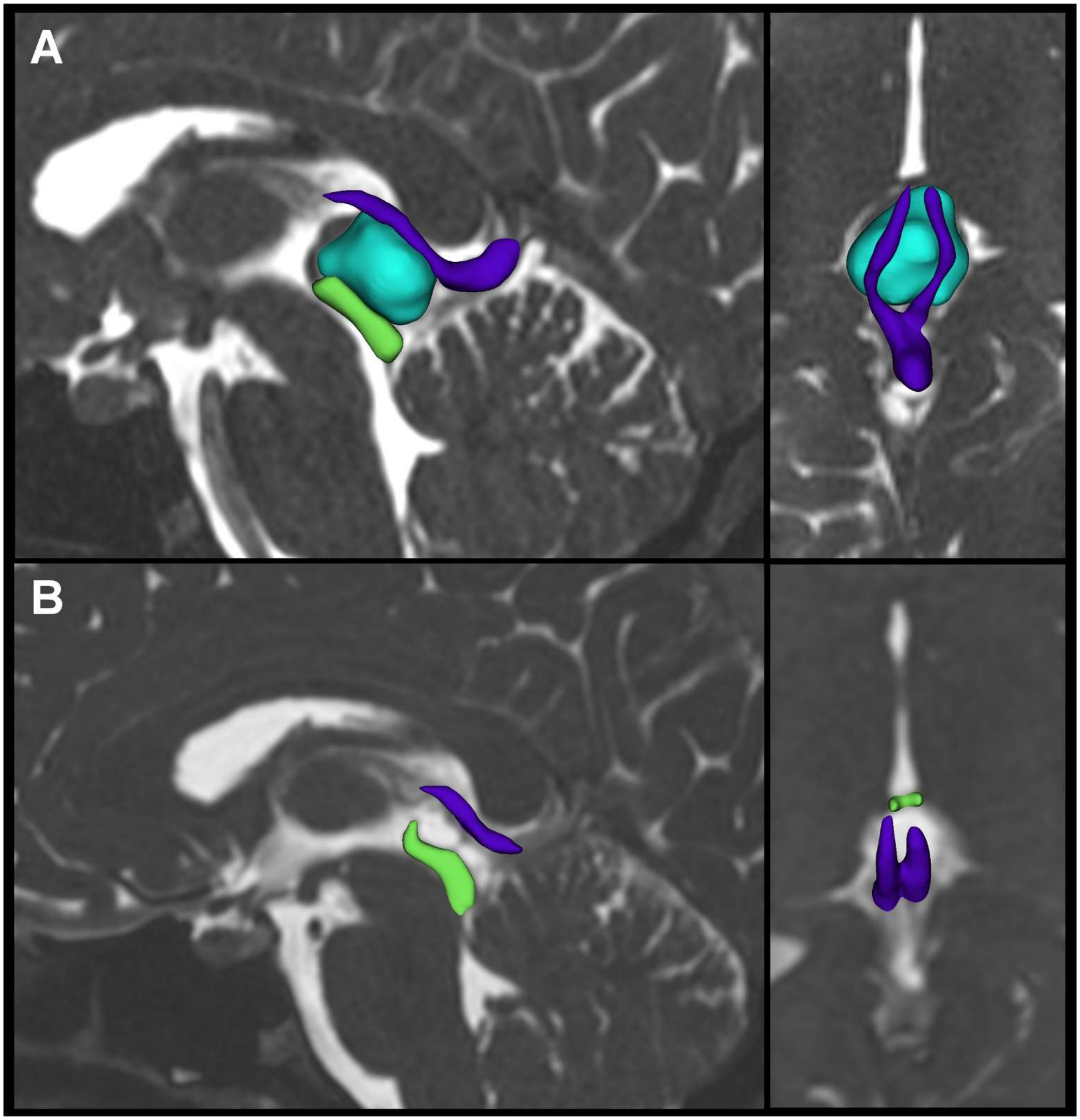

Supplementary Figure 1. (A) 3D presentation of a pineal cyst (turquoise) causing crowding of the pineal recess, occupying the space between the tectum (green) and internal cerebral veins (blue), presented in sagittal (left) and axial (right) planes. (B) 3D presentation of the pineal recess after removal of the cyst, which was accompanied with symptom relief, shown in sagittal (left) and axial (right) planes. Image: Tomas Sakinis, MD.

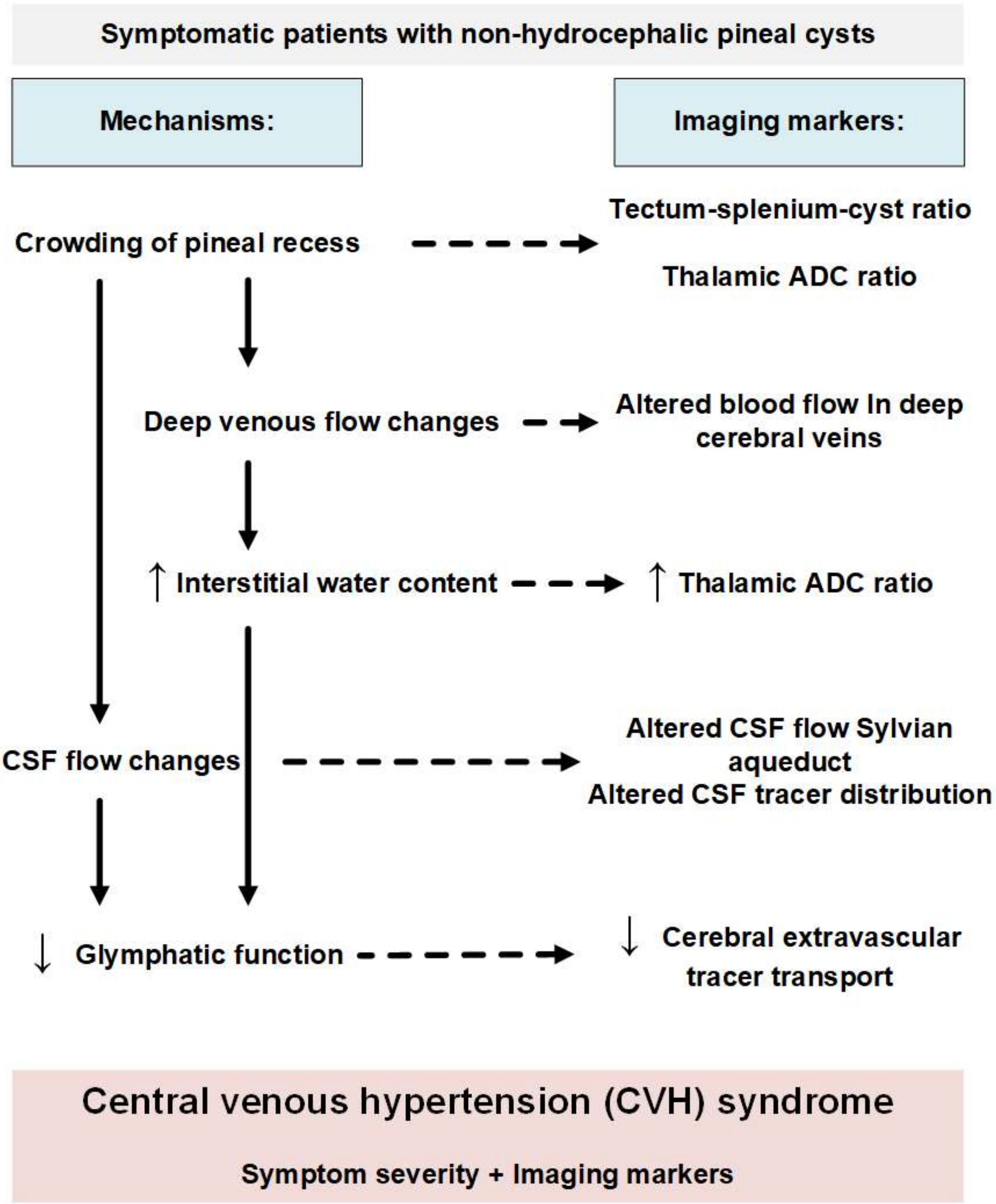

Supplementary Figure 2. A flow chart of possible pathophysiological mechanisms (left column) in symptomatic non-hydrocephalic pineal cyst patients. Previously, we referred to possible consequences of venous obstruction with subsequent interstitial water increase as a central venous hypertension syndrome.[1] Here, we report possible imaging biomarkers (right column) reflecting the tentative underlying mechanisms. This flow chart does not claim to provide a complete picture as other mechanisms most likely are at play. Rather it conceptualizes our thinking about possible events in symptomatic patients with non-hydrocephalic pineal cysts.

Supplementary Table 1. A scale for grading of symptoms in patients with pineal cysts.[1]

Symptom	Severity	Score
Headache	No	0
	Minor-Moderate	1
	Severe	2
Nausea/Vomiting	No	0
	Yes	1
Dizziness	No	0
	Yes	1
Visual disturbances	No	0
	Yes	1
Episodic loss of consciousness	No	0
	Yes	1
Lethargy/Fatigue	No-Moderate	0
	Severe	1
Cognitive impairment	No	0
	Minor-Moderate	1
	Severe	2
Transient neurological deficits	No	0
	Yes	1
**Total score**		**0/10 – 10/10**

Symptom categories:
«Minor»: 0-2/10 scores.
«Moderate» 3-4/10 scores.
«Severe» 5-10/10 scores.

Supplementary Table 2. MRI biomarkers of crowding within the pineal recess in symptomatic patients with non-hydrocephalic PCs.[1]

PC Grading	MRI biomarkers of crowding within the pineal recess
Grade 1	Tectum-splenium-cyst ratio ≤0.90<br>Thalamic ADC ratio ≤1.01
Grade 2	Tectum-splenium-cyst ratio >0.90<br>Thalamic ADC ratio ≤1.01
Grade 3	Tectum-splenium-cyst ratio ≤0.90<br>Thalamic ADC ratio >1.01
Grade 4	Tectum-splenium-cyst ratio >0.90<br>Thalamic ADC ratio >1.01

ADC: Apparent diffusion coefficient. MRI: Magnetic resonance imaging. PC: Pineal cyst

Supplementary Table 3. Estimates of venous flow in internal cerebral vein proximal to (upstream) and at the cyst

	Measures of flow - Right internal cerebral vein			Measures of flow - Left internal cerebral vein		
	**Proximal to cyst**	**At cyst**	**Change (%) (at cyst vs proximal)**	**Proximal to cyst**	**At cyst**	**Change (%) (at cyst vs proximal)**
**Maximum flow velocity (cm/s)**	7.79 ± 1.63	8.26 ± 1.79	7.18 ± 16.70	7.56 ± 1.41	8.00 ± 1.58	7.76 ± 21.59
**Mean flow velocity (cm/s)**	6.98 ± 1.47	7.48 ± 1.65	8.51 ± 18.17	6.77 ± 1.27	7.27 ± 1.44	9.10 ± 20.71
**Stroke volume (cm^3)**	0.20 ± 0.06	0.24 ± 0.08	18.43 ± 23.95	0.20 ± 0.05	0.24 ± 0.06	20.50 ± 22.08
**Region of interest (cm^3)**	0.03 ± 0.01	0.04 ± 0.01	-	0.04 ± 0.01	0.04 ± 0.01	-

Data presented as mean ± standard deviation.

Supplementary Table 4. Correlations between tracer enrichment in thalamus and tracer enrichment in CSF and brain spaces

		Tracer enrichment in thalamus	
Tracer enrichment in CSF		6 hrs	24 hrs
Cisterna magna	6 hrs	R=0.52, P=0.010	
	24 hrs		R=0.73, P<0.001
Nearby pineal cyst	6 hrs	R=-0.04, ns	
	24 hrs		R=0.50, P=0.010
Vertex	6 hrs	R=0.46, P=0.025	
	24 hrs		R=0.65, P<0.001
4th ventricle	6 hrs	R=0.24, ns	
	24 hrs		R=0.58, P=0.002
3rd ventricle	6 hrs	R=0.23, ns	
	24 hrs		R=0.30, ns
Lateral ventricles	6 hrs	R=0.20, ns	
	24 hrs		R=0.34, ns
Tracer enrichment in brain			
Cerebral cortex	6 hrs	R=0.62, P=0.001	
	24 hrs		R=0.91, P<0.001
Subcortical white matter	6 hrs	R=0.77, P<0.001	
	24 hrs		R=0.94, P<0.001

Tracer enrichment refers to percentage change in normalized MRI T1 signal unit ratios 6 and 24 hours after intrathecal tracer administration. Correlations were determined by Pearson correlation coefficients, with significance levels.

Supplementary Table 5. Comparisons of patients treated surgically or conservatively for pineal gland cysts.

	Surgery-group	Conservative-group	P-value
**N**	11	14	
Gender (F/M)	10/1	13/1	ns
Age (years)	33.9 ± 10.2	35.9 ± 11.2	ns
BMI (kg/m²)	28.0 ± 5.9	27.6 ± 5.2	ns
Symptoms prior to MRI			
Headache (No-Moderate/Severe)	1/10	4/10	ns
Nausea/Vomiting (No/Yes)	3/8	6/8	ns
Dizziness (No/Yes)	1/10	9/5	0.005
Visual disturbances (No/Yes)	1/10	6/8	ns
Episodic loss of consciousness (No/Yes)	7/4	14/0	0.014
Lethargy/Fatigue (No-Moderate/Severe)	1/10	3/11	ns
Cognitive impairment (No-Moderate/Severe)	6/5	12/2	ns
Transient neurologic deficits* (No/Yes)	2/9	8/6	ns
Grading of symptoms [1]			
Minor (n, %)		1 (7%)	P=0.045
Moderate (n, %)		5 (36%)	
Severe (n, %)	11 (100%)	8 (57%)	
Occupational ability			
Inability to work/schooling due to symptoms (n, %)	10 (91%)	9 (64%)	ns
Subjective sleep quality			
PSQI Total score	12.2 ± 3.1	8.7 ± 4.3	0.04
MRI indices of hydrocephalus			
Evans index	0.25 ± 0.03	0.26 ± 0.02	ns
Callosal angle (degrees)	119 ± 14	122 ± 10	ns
Volume 4th ventricle (ml)	1.6 ± 0.6	1.3 ± 0.4	ns
Volume 3rd ventricle (ml)	0. 9 ± 0.4	0.8 ± 0.3	ns
Volume lateral ventricles (ml)	14.0 ± 7. 7	12.5 ± 5.3	ns
MRI indices of crowded pineal recess			
Tectum-splenium-cyst ratio	0.95 ± 0.10	0.87 ± 0.16	ns
Tectum-splenium-cyst ratio >0.90 (n, %)	8 (73%)	7 (50%)	ns
Thalamic ADC ratio	1.02 ± 0.03	1.02 ± 0.04	ns
Thalamic ADC ratio >1.01 (n, %)	5/11	7/14	
Anterior-posterior diameter of cyst (mm)	17.0 ± 3.2	16.4 ± 5.4	ns
Tectum compression + Aqueduct stenosis (No/Moderate)	5/6	11/3	ns

Categorical data presented as numbers (ranges in parentheses); continuous data presented as mean ± standard deviation. Significant differences between groups were determined by Pearson Chi-square test for categorical data and by independent samples t-test for continuous data. Ns: non-significant differences between groups.

Supplementary Table 6. Comparisons of MRI measures of blood flow changes in internal cerebral vein and cerebral aqueduct between patients managed surgically or conservatively for pineal gland cysts

	Surgery-group	Conservative-group	P-value
Flow changes in internal cerebral veins proximal and at cyst (n=18)			
Change in maximum flow velocity at cyst, left (%)	12.0 ± 24.4	5.1 ± 20.3	ns
Change in maximum flow velocity at cyst, right (%)	8.2 ± 9.5	6.6 ± 20.5	ns
Change in mean flow velocity at cyst, left (%)	13.4 ± 23.7	6.4 ± 19.3	ns
Change in mean flow velocity at cyst, right (%)	9.1 ± 9.6	8.2 ± 22.5	ns
Change in stroke volume at cyst, left (%)	13.4 ± 15.0	25.0 ± 25.2	ns
Change in stroke volume at cyst, right (%)	27.6 ± 30.0	12.6 ± 18.4	ns
CSF flow changes in cerebral aqueduct (n=20)			
Antegrade-directed net flow			
N (%)	9 (60%)	6 (40%)	
Volume (ml/cycle)	0.0027 ± 0.0020	0.0030 ± 0.0013	ns
Estimated volume (L/24 hrs)	0.2378 ± 0.175	0.335 ± 0.152	ns
Retrograde-directed net flow			
N (%)	1 (20%)	4 (80%)	
Volume (ml/cycle)	0.0130	0.0023 ± 0.0015	-
Estimated volume (L/24 hrs)	1.374	0.2148 ± 0.1463	-

Supplementary Table 7. Clinical outcome in the patients treated surgically or conservatively for pineal gland cysts

	Surgery-group	Conservative-group	P-value
Outcome			
Worse	0	1	
Unchanged	0	10	P<0.001
Some improvement	1	2	
Marked improvement	10	1	
Observation period			
Years (mean±stdev)	1.9±1.2	3.1±2.1	ns

Supplementary Table 8. Enrichment of tracer within the main brain regions of patients treated surgically (SURG) or conservatively (CONS) for pineal gland cysts.

Anatomical region	Group	0.5 – 2 hours				2 - 4 hours				4 - 7 hours				~24 hours				~48 hours			
		Mean	±	SE	p	Mean	±	SE	p	Mean	±	SE	p	Mean	±	SE	p	Mean	±	SE	p
Frontal lobe (GM)	CONS	3	±	1	ns	55	±	3	<0.001	127	±	4	<0.001	117	±	3	<0.001	47	±	2	ns
	SURG	4	±	1		36	±	3		104	±	4		99	±	3		48	±	2	
Frontal lobe (WM)	CONS	-2	±	1	ns	0	±	1	ns	8	±	1	ns	38	±	2	0.015	25	±	1	ns
	SURG	-2	±	1		-2	±	1		6	±	1		33	±	2		23	±	1	
Temporal lobe (GM)	CONS	5	±	2	ns	65	±	5	0.02	126	±	5	0.004	98	±	4	0.011	42	±	2	ns
	SURG	9	±	2		49	±	5		102	±	6		83	±	5		44	±	3	
Temporal lobe (WM)	CONS	-3	±	1	ns	2	±	1	ns	14	±	2	ns	41	±	2	ns	26	±	2	ns
	SURG	0	±	1		2	±	1		12	±	2		36	±	3		26	±	2	
Parietal lobe (GM)	CONS	2	±	1	ns	37	±	3	0.005	94	±	4	<0.001	99	±	4	<0.001	44	±	2	ns
	SURG	2	±	1		24	±	4		62	±	4		75	±	4		42	±	2	
Parietal lobe (WM)	CONS	-2	±	1	ns	0	±	1	ns	8	±	1	0.025	32	±	2	0.001	22	±	1	ns
	SURG	-1	±	1		-1	±	1		5	±	1		24	±	2		19	±	2	
Occipital lobe (GM)	CONS	5	±	1	ns	35	±	4	0.047	76	±	5	<0.001	75	±	4	0.001	35	±	2	0.015
	SURG	4	±	2		22	±	5		49	±	6		53	±	5		27	±	3	
Occipital lobe (WM)	CONS	-1	±	1	ns	1	±	1	ns	10	±	1	0.022	30	±	2	0.019	19	±	1	ns
	SURG	-3	±	1		-2	±	1		6	±	2		22	±	3		14	±	2	
Cingulate cortex (GM)	CONS	11	±	1	ns	77	±	5	0.001	158	±	5	<0.001	98	±	4	0.004	38	±	2	ns
	SURG	8	±	1		55	±	5		123	±	6		80	±	5		37	±	2	
Cingulate cortex (WM)	CONS	0	±	1	ns	2	±	1	ns	9	±	1	ns	26	±	2	ns	15	±	1	ns
	SURG	-1	±	1		0	±	1		6	±	1		23	±	2		14	±	1	

GM: Gray matter; WM: White matter. Data presented as percentage change in normalized T1 signal units over time. P: p-value (mixed model analysis); ns: non-significant differences between groups.

References

1. Eide PK, Pripp AH, Ringstad GA. Magnetic resonance imaging biomarkers indicate a central venous hypertension syndrome in patients with symptomatic pineal cysts. *J Neurol Sci*. Apr 15 2016;363:207-16. doi:10.1016/j.jns.2016.02.038
